# Time‐of‐day, satellite cells, and velocity collectively influence ex vivo isovelocity force production in mouse extensor digitorum longus muscle

**DOI:** 10.14814/phy2.70902

**Published:** 2026-05-03

**Authors:** Ryan E. Kahn, Sudarshan Dayanidhi, Richard L. Lieber

**Affiliations:** ^1^ Shirley Ryan AbilityLab Chicago Illinois USA; ^2^ Feinberg School of Medicine Northwestern University Chicago Illinois USA; ^3^ Hines VA Medical Center Maywood Illinois USA

**Keywords:** force production, force‐velocity, muscle mechanics, satellite cells, time‐of‐day

## Abstract

Skeletal muscles are exquisitely designed to produce force that facilitate movement. Circadian “molecular clocks” residing in muscle play a role in regulating force production with muscle stem cell (*satellite cells*, *SC)* molecular clocks modulating isometric and eccentric force according to time‐of‐day. However, many tasks of daily living and exercise (i.e., walking/running) involve force and power produced during muscle shortening. Thus, the purpose of this study was to determine whether isovelocity forces and power are also modulated by SCs according to time‐of‐day. Using previously published samples (a mouse model capable of SC ablation), we evaluated isovelocity forces across a range of velocities (1–11 L_f_/s) at two different times of day ZT1, ZT9 in the presence and absence of SCs. The main finding of this investigation was that isovelocity force production is regulated by a third‐order interaction effect between time‐of‐day × SCs × velocity (*p* < 0.001). Additionally, a significant effect of time‐of‐day was observed for isovelocity force and power when comparing ZT1 vs. ZT9 SC^+^ mice whereas this effect was absent in SC^−^ animals. These results suggest SCs harbor a time‐of‐day and velocity dependent effect on isovelocity force production and power. Further work is required to elucidate the underlying mechanisms of this phenomenon.

## 1 INTRODUCTION

Skeletal muscles comprise approximately 40% of body mass and are responsible for producing forces that facilitate activities such as exercise (Smith et al., 2023). Exercise performance is a circadian regulated activity (Adamovich et al., 2021; Ezagouri et al., 2019) partially attributed to “molecular clock” influences that, in turn, affect force production (Douglas et al., 2021). Molecular clocks residing in skeletal muscle have been shown to regulate isometric force as evidenced by whole‐body and muscle‐specific molecular clock‐knockout (KO) mouse models displaying reduced isometric force production (Andrews et al., 2010; Dyar et al., 2014; Kumar et al., 2024; Schroder et al., 2015). Further, human evidence has shown a diurnal rhythmicity to voluntary isometric torque (Ab Malik et al., 2020; Douglas et al., 2021; Reilly & Brooks, 1982).

In addition to muscle molecular clocks, the muscle stem cell population (“satellite cells,” SC) also house oscillating molecular clocks (Solanas et al., 2017) that may play a role in regulating contractile force production (Kahn et al., 2024; Kahn, Dinnunhan, et al., 2025; Kahn, Zhu, et al., 2025). Circadian expression of SC molecular clock and contractile‐related genes peaks/troughs (Solanas et al., 2017) was recently demonstrated to be aligned with highs/lows in isometric torque output (Zhu et al., 2025). Furthermore, our prior work demonstrated that SCs differentially regulate isometric and eccentric forces according to time‐of‐day (Kahn, Zhu, et al., 2025). Confirming direct regulation of SC molecular clocks on force production, we recently showed that SC‐specific ablation of clock‐gene, *Bmal1*, reduced isometric and eccentric forces both in vivo and ex vivo and altered the repair response after contractile‐induced injury (Kahn, Zhu, et al., 2025).

While this evidence indicates SC molecular clocks influence isometric and eccentric forces according to time‐of‐day (Kahn et al., 2024; Kahn, Zhu, et al., 2025; Zhu et al., 2025), forces produced during typical locomotion and exercise involve dynamic shortening such as isovelocity contractions. In this regard, it has previously been shown that exercise performance is a time‐of‐day regulated event (Adamovich et al., 2021; Ezagouri et al., 2019; Maier et al., 2022). While these reports (Adamovich et al., 2021; Ezagouri et al., 2019; Maier et al., 2022) and others (Kahn, Dinnunhan, et al., 2025) have shown alterations in exercise performance and contractile fatigue may stem from molecular clock time‐of‐day effects on muscle metabolism, it remains unknown whether isovelocity contractile properties are also under time‐of‐day influence. Assessing in vivo isovelocity force during exercise is unfortunately limited in experimental techniques; however, ex vivo approaches of assessing isovelocity force have a rich history in muscle physiology (Brooks et al., 1990; Brooks & Faulkner, 1988; Claflin & Faulkner, 1985; Curtin & Edman, 1994; Hill, 1953; Lutz et al., 2002; Lutz & Lieber, 2000). Therefore, the purpose of this study was to determine whether isovelocity forces and power are also modulated by SCs according to time‐of‐day. Using previously published samples (a mouse model capable of SC ablation), we evaluated isovelocity forces across a range of velocities (1–11 L_f_/s) at two different times of day: ZT1, ZT9 in the presence and absence of SCs.

## 2 METHODS

### 2.1 Animal model

Pax7^CreERT2/+^; Rosa26^DTA/+^ mice of mixed sex (*n* = 20; 10 M, 10 F), ages 4–6 months (Jackson Laboratories, Bar Harbor, ME, stock numbers 017763 and 010527, respectively) were used for all experiments (Fry et al., 2015; Kinney et al., 2017; McCarthy et al., 2011; Murach et al., 2017). These animals were used in our previous work (Kahn et al., 2024). In (Kahn et al., 2024), this work focused solely on isometric and eccentric properties, as well as contractile injury. The current report focuses exclusively on the force‐velocity and power properties assessed in these same mice. The full experimental timeline per muscle consisted of three protocols: three isometric contractions separated by 3 min rest each, 11 isovelocity contractions separated by 2 min rest each, and 10 eccentric contractions separated by 3 min rest each. The time each muscle was mounted for contractile testing was ~60 min. For relevance to this report, force‐velocity contractions were commenced approximately 10 min after muscle was mounted. All animal experiments were performed with the approval of the Northwestern University Institutional Animal Care and Use Committee (protocol number: IS000019422). All procedures were conducted in accordance with institutional guidelines and applicable regulations for the ethical care and use of laboratory animals. Every effort was made to minimize animal discomfort and to reduce the number of animals used. Mice had ad libitum access to food/water and were housed on a 14:10 light–dark cycle (lights‐on at 0600 h) (LabDiet (Purina), PICO‐VAC Mouse Diet 20 Irradiated, Cat# 6955). Mice were euthanized via CO2 inhalation followed by cervical dislocation. Experiments were carried out at 0700 h (ZT1) and at 1500 h (ZT9). At either timepoint, only one experiment was performed on an experimental day. Our previous work shows rhythmic oscillation of molecular clock gene expression under a 14:10 light–dark photoperiod (Kahn et al., 2024; Kahn, Dinnunhan, et al., 2025). As previously described, inducible depletion of SCs was carried out via Cre‐Lox mediated Pax7^+^ cell ablation involving five consecutive days of oral gavage of either tamoxifen (2 mg/mL) or vehicle (peanut oil) followed by a 10‐day washout period (Kahn et al., 2024; Kinney et al., 2017). After these treatments, mice were evaluated in ZT1 and ZT9 SC^+^ and SC^−^ experimental groups. Experiments were performed after euthanasia at each timepoint. These same animals were used in our previous work where we quantified SC ablation (Kahn et al., 2024).

### 2.2 Muscle isolation and experimental apparatus

Briefly, the EDL of the left hindlimb was isolated and quickly mounted between a force transducer (Aurora 300C, Aurora Scientific, Ontario, Canada) and length motor in a custom bath of Ringer's solution at 37°C with platinum electrodes straddling the muscle, as previously described (Chapman et al., 2014; Palmisano et al., 2015; Sam et al., 2000). Force, length, and time were recorded on both an oscilloscope and a custom LabVIEW program. Twitch contractions were used to optimize muscle length/voltage; muscle length was measured, and fiber length was subsequently calculated using a standard fiber‐length‐to‐muscle‐length ratio of 0.51 for the EDL (Burkholder et al., 1994). These stimulation parameters were adopted from previous studies (Chapman et al., 2014; Kahn et al., 2024; Kahn, Dinnunhan, et al., 2025; Kahn, Zhu, et al., 2025; Palmisano et al., 2015; Sam et al., 2000). Force was recorded in volts, based on a calibration curve, and converted to Newtons (N). This was expressed as specific force (N/cm^2^) by normalizing to physiological cross‐sectional area (PCSA). PCSA was calculated using the following equation:

$$\text{PCSA}=M/\left[L_{o}\times \left(L_{f}/L_{o}\right)\times \rho \;\right]$$

where *M* is mass, *L*_o_ is optimal muscle length, L_f_ is optimal fiber length, and *ρ* is fiber density assumed to be 1.056 g/cm^3^ for skeletal muscle (Méndez & Keys, 2026). After experimentation, muscles were unmounted, blotted dry, weighed, and flash frozen in liquid nitrogen‐cooled isopentane for storage at −80°C.

### 2.3 Force‐velocity experiment

**FIGURE 1:**
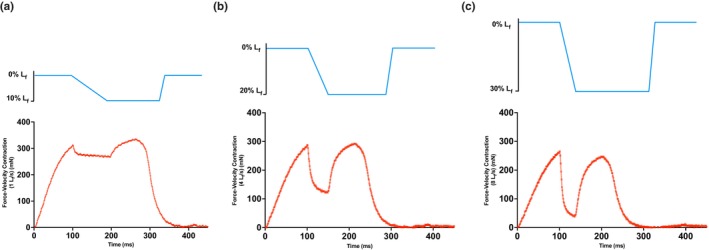
Force‐velocity contractile traces (a–c) Representative force (red) and length (blue) traces of isovelocity shortening contractions at 1, 4, 8 L_f_/s where all contractions were initiated by stimulating for 100 ms isometrically before the shortening ramp at the appropriate predetermined velocity, and regained isometric tension for the remainder of the contraction. The rapid decline in force toward the end of the force trace is when stimulation was terminated.

A family of force‐velocity (isovelocity) curves was generated using previously published methods with minor modifications (Brooks et al., 1990; Brooks & Faulkner, 1988; Claflin & Faulkner, 1985; Lutz et al., 2002). Eleven isovelocity contractions were elicited with 2 min rests between contractions to avoid excessive fatigue. During each contraction, the muscle was stimulated isometrically for 100 ms and then shortened by a pre‐set length change at a pre‐set velocity. Immediately prior to shortening, a very brief supra‐physiological shortening was imposed on the muscle to release series‐elasticity before the force‐velocity contraction (Lutz et al., 2002). Isovelocity contractions were performed across velocities of 1–11 L_f_/s, with muscle shortening by 10%, 20%, and 30% L_f_ for 1–2 L_f_/s, 3–6 L_f_/s, and 7–11 L_f_/s, respectively. After shortening contractions, muscles redeveloped tension until termination of stimulation. At each shortening velocity, muscle force decreased exponentially until it stabilized for a brief period of time (Figure 1). Force maintained in this brief period of time was defined as the force generated at that particular shortening velocity. Force values across isovelocity contractions 1–11 L_f_/s were then compared across groups.

**FIGURE 2:**
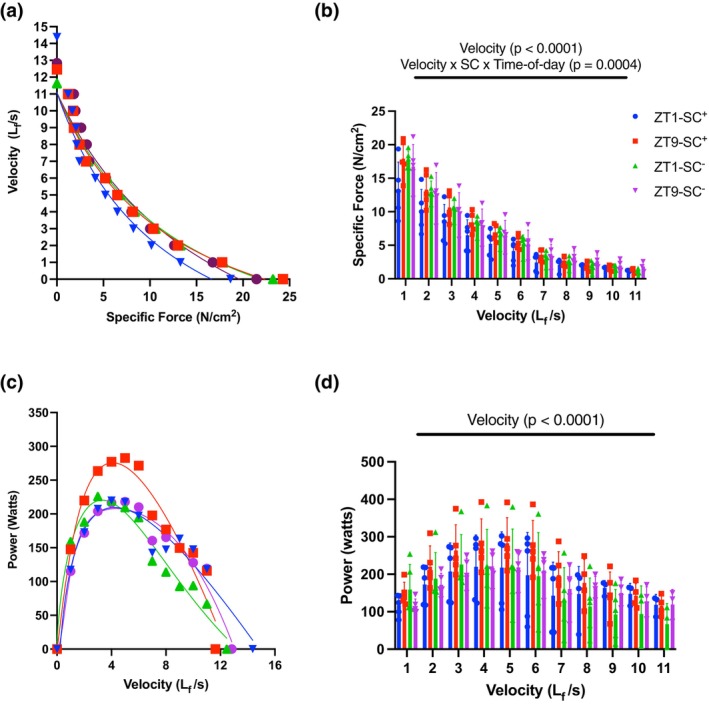
Isovelocity forces and power produced during shortening contractions (a) Force‐velocity curves with force expressed in units of specific force (N/cm^2^) and velocity (L_f_/s). Each data point of the force‐velocity curves correspond to the force produced at each respective shortening velocity (b) Isovelocity force produced by each group from velocities 1–11 L_f_/s (Velocity: *p* < 0.0001), (Velocity × SC × Time‐of‐day: *p* = 0.0004). (c) Power curves with power expressed in units of watts and velocity as (L_f_/s). Each data point on the power curve corresponds to the power produced at each respective shortening velocity (d) Power produced by all groups from velocities 1–11 L_f_/s (velocity: *p* < 0.0001). All data shown as mean ± SD. All groups compared via three‐way ANOVA (*n* = 5 mouse EDLs per group).

Nonlinear regression of the hyperbolic Hill equation form (P + a) (V + b) = (P_o_ + a) b, expressed as V = (P_o_ − P)b/(P + a), (where P_o_ is maximal tetanic specific force, P is specific force during a single force‐velocity contraction, and V is the velocity of a single contraction) was used on raw force‐velocity data to calculate coefficients a and b (Hill, 1938). Extracted coefficients, a and b, were used to calculate maximum shortening velocity, *V*_max_ (*V*_max_ = P_o_b/a) (Claflin & Faulkner, 1985; Fry et al., 2015; Hill, 1953; Lutz et al., 2002; Mayeuf‐Louchart et al., 2018) which was compared across groups. Power was calculated by multiplying force (mN) by velocity (mm/s) values and reported in units of watts. Force‐velocity and power curves (Figure 2a, Figure 2c) were fit on average data and are a representative trace for each group. Analysis of isovelocity force and power across velocities is displayed in Figure 2b,d. Work was calculated by multiplying the distance fibers shortened (mm) by specific force (N/cm^2^). Total force‐loss was calculated as the percentage of force‐loss from the tetanic contraction immediately before and after isovelocity contractions.

### 2.4 Immunohistochemistry (IHC)

EDL muscles were flash frozen and stored at −80°C. Each muscle was transferred to a cryostat set to −25°C to section for immunohistochemical staining of fiber‐types (2A, 2B) and myofiber borders (laminin) as previously described (Kahn et al., 2023, 2024; Kahn, Zhu, et al., 2025). IHC slides were imaged at 10× and whole tilescan cross‐section images were analyzed for cross‐sectional area and fiber‐type composition (MHC 2A, 2B) through the automated quantification/analysis software, MuscleJ (Mayeuf‐Louchart et al., 2018), and any unlabeled fibers were considered to be 2× fibers. A total of four cross‐sections were placed on each slide; one section was selected for imaging and analysis per slide. Primary antibody details were as follows: anti‐laminin (rabbit IgG, 1:500, Sigma‐Aldrich, L9393), anti‐SC71 (mouse, IgG1, 1:50, lot # 2147165, DSHB, Iowa City, IA, USA), anti‐BF‐F3 (mouse, IgM, 1:100, lot # 2266724, DSHB, Iowa City, IA, USA). Secondary antibody details were as follows: Alexa Fluor 488 goat anti‐rabbit IgG (H + L) (1:250, Invitrogen, A‐11034), Alexa Flour 488 goat anti‐mouse IgG1 (1:250, A21121, Invitrogen, Waltham, MA, USA), goat anti‐mouse IgM (1:250, A‐21426, Invitrogen, Waltham, MA, USA).

### 2.5 Statistical analyses

All data were analyzed by three‐way or two‐way ANOVA, as appropriate to the experimental design. A three‐way ANOVA experimental design with time‐of‐day, presence of SCs, and contraction velocity as grouping factors was used to analyze our force‐velocity and power data. Velocity was the only repeated measure variable. Two‐way ANOVA experimental designs were used to analyze force‐velocity and power data when assessing time‐of‐day groups (ZT1 vs. ZT9) and total work, force‐loss. Specific statistical tests are indicated in figure legends. All statistical analyses were performed using Prism 9.0 (GraphPad, San Diego, CA). All data in the results are reported as means ± standard deviation (SD). Significance level (a) was set to 0.05 for all parametric tests.

## 3 RESULTS

### 3.1 Isovelocity forces and power produced during shortening contractions across all groups (three‐way‐ANOVA)

Isovelocity force and power were measured across time‐of‐day and in the presence/absence of SCs to evaluate the collective influence from SCs, velocity, and time‐of‐day. As expected, based on the classic muscle force‐velocity relationship, a main effect of velocity was observed for isovelocity force and power generated across all velocities (*p* < 0.0001; Figure 2b,d), reflecting the intrinsic isovelocity force generating properties of skeletal muscle (Hill, 1953). A highly significant third‐order interaction was observed between velocity × SC × time‐of‐day in the isovelocity force data (*p* < 0.001; Figure 2b); however, this was not observed in the power data (Figure 2d). The main finding of this section was that isovelocity force was affected by a third‐order interaction effect between velocity × SCs × time‐of‐day.

### 3.2 Time‐of‐day specific comparison of isovelocity forces and power produced during shortening contractions (two‐way‐ANOVA)

**FIGURE 3:**
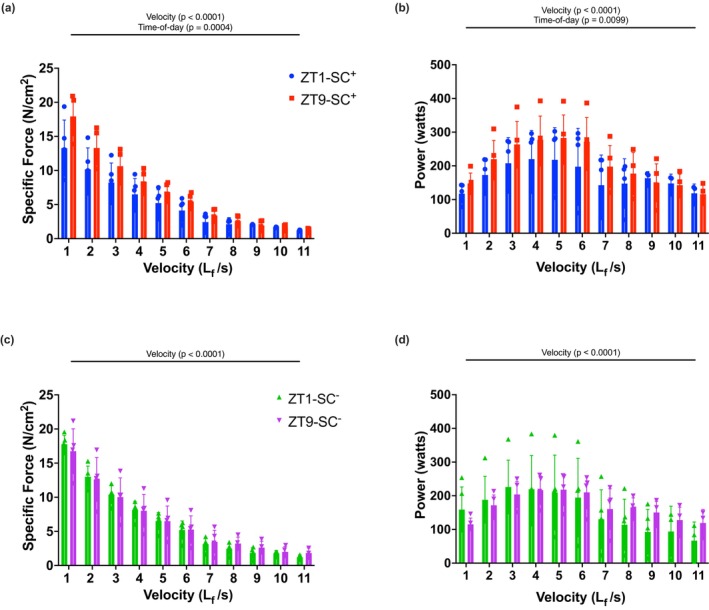
Time‐of‐day specific comparisons of isovelocity and power (a) Isovelocity forces produced by SC^+^ groups from velocities 1–11 L_f_/s (velocity: *p* < 0.0001), (time‐of‐day: P = 0.0004). (b) Power produced by SC^+^ groups from velocities 1–11 L_f_/s (velocity: *p* < 0.0001), (time‐of‐day: *p* = 0.0099). (c) Isovelocity forces produced by SC^−^ groups from velocities 1–11 L_f_/s (velocity: *p* < 0.0001). (d) Power produced by SC^−^ groups from velocities 1–11 L_f_/s (velocity: *p* < 0.0001). All data shown as mean ± SD. All groups were compared via two‐way ANOVA (*n* = 5 mouse EDLs per group).

While the previous results section evaluated the relationship of all three experimental variables (velocity, SCs, time‐of‐day), the purpose of this section was to analyze the SC^+^ and SC^−^ groups separately to evaluate the effect of time‐of‐day on isovelocity force and power. A main effect of velocity was observed for isovelocity force and power generated across all velocities in both Moring versus ZT9 SC^+^ and SC^−^ groups (*p* < 0.0001; Figure 3a–d). A significant effect of time‐of‐day was observed when comparing isovelocity force and power in ZT1‐SC^+^ versus ZT9‐SC^+^ animals (*p* < 0.001; Figure 3a) (*p* < 0.01; Figure 3b). No time‐of‐day effect was observed in ZT1 versus ZT9 SC^−^ groups (Figure 3c,d). The main finding of this section was that a time‐of‐day effect on isovelocity force and power was observed in SC^+^ mice from ZT1 to ZT9 whereas this effect was not observed in SC^−^ animals.

### 3.3 Contractile characteristics

Here we evaluated contractile characteristics that could help explain the main findings in the abovementioned results sections. No significant differences in total work were observed among groups (ZT1‐SC^+^: 80 ± 31 ZT9‐SC^+^: 111 ± 19; ZT1‐SC^−^: 111 ± 11; ZT9‐SC^−^: 105 ± 25; (N/cm^2^ × mm)). Total force‐loss following all force‐velocity contractions was similar between groups (ZT1‐SC^+^: 33 ± 13 ZT9‐SC^+^: 29 ± 7; ZT1‐SC^−^: 30 ± 9; ZT9‐SC^−^: 22 ± 9; (%force loss)). *V*_max_ was similar across groups (ZT1‐SC^+^: 14 ± 3.7; ZT9‐SC^+^: 12 ± 1; ZT1‐SC^−^: 13 ± 3; ZT9‐SC^−^: 12 ± 2; (L_f_/s)). Overall, no differences in contractile characteristics were observed.

As this study used both male and female mice which can differ in BW, muscle weight, and raw force, we normalized force to PCSA (specific force) to accurately present force data from mixed sexes. Figure S1 illustrates this showing a main effect of sex in BW (*p* < 0.0001), EDL weight (*p* < 0.001), and raw forces (*p* < 0.05) while no significant differences were observed on specific force in male versus female mice. Force in millinewtons and specific force shown in Figure S1 are from isometric maximal force measurements.

### 3.4 Satellite cell ablation and myofiber characteristics

**FIGURE 4:**
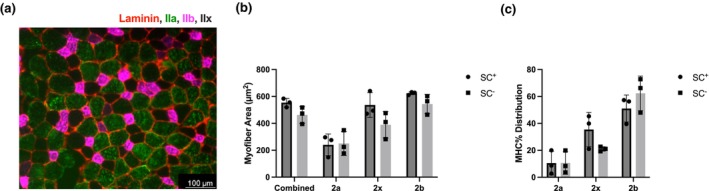
Myofiber area and fiber‐type composition (a) Representative image of an EDL cross‐section displaying 2A, 2X, and 2B fibers (pink, black/unlabeled, green, respectively). (b) Myofiber area by fiber‐type. (c) MHC fiber‐type distribution. All data shown as mean ± SD. All groups compared by two‐way ANOVA (*p* > 0.05) (*n* = 3 mouse muscles per group).

SC (Pax7) abundance, previously reported (Kahn et al., 2024), was significantly reduced (~75% reduction) after tamoxifen‐induced ablation. No baseline differences in myofiber area or fiber‐type composition (MHC% distribution) were observed (Figure 4a–c).

## 4 DISCUSSION

The purpose of this paper was to determine whether SCs differentially modulate isovelocity contractile force depending on time‐of‐day and presence of SCs. The main finding of this investigation was that isovelocity force production was not simply a function of time‐of‐day or SC presence, but was regulated by a third‐order interaction effect between time‐of‐day × SCs × velocity. When comparing time‐of‐day groups to each other, a significant effect of time‐of‐day was observed for isovelocity force and power produced between ZT1‐SC^+^ versus ZT9‐SC^+^; however, this was not observed in ZT1 versus ZT9 SC^−^ groups. Across SC^+^/SC^−^ ZT1 and ZT9 groups, there were no differences in total work, force‐loss, *V*_max_, or fiber‐type. These results demonstrate that SCs harbor a time‐of‐day and velocity dependent effect on isovelocity force production.

This work provides additional observations from mice previously studied for isometric and eccentric properties as well as contractile injury (Kahn et al., 2024). It does not address reproducibility of the prior study. The additional observations demonstrate isovelocity force and power are perhaps under SC time‐of‐day‐dependent regulation and the relationship may be velocity dependent, in this set of mice. Interestingly, in our previous work we did not observe a time‐of‐day effect on isometric or eccentric force suggesting isovelocity force and power may be under differential regulation versus isometric and eccentric forces. The physiological meaning or mechanistic underpinning of this relationship is beyond the scope of this short report; however, our preliminary observation of the existence of this relationship is most intriguing.

Additionally, while the underlying mechanism is unclear, the application of these findings is relevant in the contexts of SC and time‐of‐day regulations on exercise performance. Exercise performance has been demonstrated to be under robust time‐of‐day control (Adamovich et al., 2021; Ezagouri et al., 2019; Kemler et al., 2020; Maier et al., 2022; Wolff & Esser, 2012) however, the relevance of SCs on these time‐of‐day regulations has not been investigated. In this regard, prior evidence suggests that time‐of‐day effects on muscle metabolism may be an underlying mechanism explaining diurnal exercise performance (Adamovich et al., 2021; Ezagouri et al., 2019). We've previously shown that SCs had a time‐of‐day influence on mitochondrial metabolism which may have influenced divergent rates of contractile fatigue in that report (Kahn, Dinnunhan, et al., 2025). In addition to this prior evidence, this report now reveals that isovelocity contractile properties are also under time‐of‐day influence. Isovelocity forces are mimetic of the shortening contractions produced during running exercise (the method of exercise‐capacity assessment in the referenced studies (Adamovich et al., 2021; Ezagouri et al., 2019; Kemler et al., 2020; Maier et al., 2022; Wolff & Esser, 2012)) and therefore are relevant in these contexts of exercise performance. Collectively, the evidence from these combined works suggests that previous observations of diurnal trends in exercise performance may be related to SC time‐of‐day effects on muscle metabolism (Kahn, Dinnunhan, et al., 2025) and isovelocity properties.

In the contexts of past work on these mice in Kahn et al. (2024), we had not previously performed three‐way or two‐way ANOVAS on isovelocity force and power values across velocities as the comparisons we were interested in involved only two main independent variables (time‐of‐day and SCs) and velocity had not been considered. However, upon a reexamination of this data set with a more nuanced approach, we have made surprising observations that isovelocity force and power are under SC time‐of‐day regulations with the effect on isovelocity force being velocity‐dependent. While the data presented in this report are observational in nature, we believe it may highlight the nuance of time‐of‐day regulations on muscle physiology and warrant further exploration to elucidate underlying mechanisms.

In this work we show no difference in force‐loss following isovelocity contractions. While force‐loss is not a direct reflection of fatigue, it is worth noting that this data aligns with prior work showing that fatigue induced by maximal ex vivo contractions does not exhibit time‐of‐day variance (Fitzgerald et al., 2024) whereas fatigue from submaximal contractions (reliant on mitochondrial energy) does exhibit time‐of‐day differences (Kahn, Dinnunhan, et al., 2025). Importantly, the force‐loss that accrues during traditional fatiguing protocols happens on a much faster timescale as muscle rests for only seconds between fatiguing contractions (Lännergren & Westerblad, 1991) whereas muscle rested for 2 min between contractions in this work. Therefore, the mechanism of force‐loss across protocols may differ (Allen et al., 2008; Lännergren & Westerblad, 1991) and thus interpretations should be cautioned in this regard. The referenced findings from past (Fitzgerald et al., 2024; Kahn, Dinnunhan, et al., 2025) and this current report highlight the complexity by which time‐of‐day regulates muscle physiology.

In the contexts of other works evaluating the relationship between SC molecular clocks and contractility, Zhu and colleagues (Zhu et al., 2025) have recently demonstrated isometric torque fluctuates in line with the diurnal nature of the SC molecular clock. Our work supports these findings suggesting the SC molecular clock may wield similar time‐of‐day regulations over *isovelocity* forces as well. Additionally, to our surprise, this effect on isovelocity force appeared to hinge on shortening velocity as well. Speculatively, SC molecular clock gene, *Bmal1*, may be most influential in the observed time‐of‐day effects on contractility. Supportive of this, inducible depletion of SC molecular clock gene, *Bmal1*, led to marked reductions in isometric and eccentric forces (Kahn, Zhu, et al., 2025) and assessments of isometric torque in line with peak/trough SC *Bmal1* expression displayed time‐of‐day differences as well (Zhu et al., 2025).

Previously, we suggested that the underlying mechanism explaining SC time‐of‐day alterations in isometric force was due to differences in SR calcium volume or availability to contractile units (Kahn et al., 2024). As the animals in the present study were also used in our previous work (Kahn et al., 2024), it is plausible that calcium may underpin differences noted here as well. Previously, elegant work has shown that calcium transients (reflective of release/sequestering) during cross‐bridge cycling influence contractile shortening velocity (Rome, 2006). In these contexts, differences in calcium transients may underpin the velocity‐dependent nature of the effects observed in this work. Supporting this notion, previous reports have shown molecular clocks indeed harbor regulation over calcium‐related contractile proteins and signaling pathways (McCarthy et al., 2007; Miller et al., 2007; Small et al., 2020).

While not specific to time‐of‐day, an intriguing finding from this work and our past works (Kahn et al., 2024; Kahn, Dinnunhan, et al., 2025) has been the observation that SCs can harbor a relationship with force production. It is well beyond the scope of this short report to suggest or even speculate on an underlying mechanism as our evidence presented here and in our past works is still early stage. Briefly however, it is interesting to consider our findings in the contexts of other recent evidence that has shown SCs are mechanically “in‐tuned” with their host myofibers (Kann et al., 2022). In these contexts, evidence has shown that SCs undergo the same degree of strain as their host myofibers when subject to ex vivo stretch (Haroon et al., 2021), SCs harbor mechanically‐sensitive protrusions that respond to injury and mechanical stretch by undergoing rapid shape‐conformational changes (Kann et al., 2022), and active cellular communication channels exist between SCs and host myofibers (Tavi et al., 2010). It is unclear if the evidence mentioned is related to our findings linking SCs with force production; however, it is a fascinating line of inquiry.

We acknowledge several limitations of this work. In this report, while we attribute time‐of‐day findings to be regulated, in part, by SC molecular clocks, our mouse model ablates the entire SC and therefore it cannot be entirely ruled out that another cellular mechanism in SCs may be contributing to our findings here. Additionally, we recognize that our sample size is low in terms of statistical power, and therefore, we've elected to present these findings as observational data within a short report format. Additionally, all forces were analyzed in units of specific force as normalizing force to PCSA accounts for differences in muscle weight, fiber length, and density that may exist between male and female mice (Glenmark et al., 2004; Hill et al., 2020) (Figure S1A–D). While our study was time‐of‐day focused, the experimental timepoints fell within the inactive/rest period; this was by design as these timepoints roughly aligned to a peak and trough in SC molecular clock transcriptional profiles (Solanas et al., 2017). Lastly, tamoxifen may have unintended metabolic effects on various organ systems and although we utilize a washout period to avoid any such effects, we cannot entirely discount the possibility of prolonged biological activity.

Collectively, this work extends our previous findings to show that, in addition to isometric and eccentric forces, isovelocity forces and power are also subject to SC time‐of‐day regulation. Surprisingly, this regulation of isovelocity force is represented by a third‐order interaction effect between SCs, time‐of‐day, and shortening velocity. More comprehensive work is required to elucidate the underlying cellular mechanisms explaining how isovelocity force and power are collectively regulated by SCs, time‐of‐day, and shortening velocity.

## AUTHOR CONTRIBUTIONS

**Ryan E. Kahn:** Conceptualization; data curation; formal analysis; investigation; project administration. **Sudarshan Dayanidhi:** Conceptualization; data curation; formal analysis; funding acquisition; investigation; methodology; project administration; resources. **Richard L. Lieber:** Conceptualization; data curation; formal analysis; funding acquisition; investigation; methodology; project administration; resources; supervision; validation; visualization.

## FUNDING INFORMATION

No funding information provided.

## CONFLICT OF INTEREST STATEMENT

The authors declare no conflicts of interest.

## ETHICS STATEMENT

All procedures were conducted in accordance with institutional guidelines and applicable regulations for the ethical care and use of laboratory animals. Every effort was made to minimize animal suffering and to reduce the number of animals used.

## Supporting information


**Figure S1.** Sex comparisons of BW, EDL weight, force, and specific force. (A–D) Sex comparisons in BW (*p* < 0.0001), EDL weight (*p* = 0.0002), force (*p* = 0.0109), and specific force (NS) in both SC^+^ and SC^−^ groups. All data shown as mean ± SD. All groups compared via two‐way ANOVA (*n* = 3–5 mouse EDLs per group).

## Data Availability

All data used within the results and to create figures are included in the material of this manuscript. Additional analysis and files can be provided upon request.

## References

[phy270902-bib-0001] Ab Malik, Z. , Bowden Davies, K. A. , Hall, E. C. R. , Barrett, J. , Pullinger, S. A. , Erskine, R. M. , Shepherd, S. O. , Iqbal, Z. , Edwards, B. J. , & Burniston, J. G. (2020). Diurnal differences in human muscle isometric force in vivo are associated with differential phosphorylation of Sarcomeric M‐band proteins. PRO, 8, 22. 10.3390/proteomes8030022 PMC7565642 32859009

[phy270902-bib-0002] Adamovich, Y. , Dandavate, V. , Ezagouri, S. , Manella, G. , Zwighaft, Z. , Sobel, J. , Kuperman, Y. , Golik, M. , Auerbach, A. , Itkin, M. , Malitsky, S. , & Asher, G. (2021). Clock proteins and training modify exercise capacity in a daytime‐dependent manner. Proceedings of the National Academy of Sciences of the United States of America, 118, e2101115118. 10.1073/pnas.2101115118 34426495 PMC8536342

[phy270902-bib-0003] Allen, D. G. , Lamb, G. D. , & Westerblad, H. (2008). Skeletal muscle fatigue: cellular mechanisms. Physiological Reviews, 88, 287–332. 10.1152/physrev.00015.2007 18195089

[phy270902-bib-0004] Andrews, J. L. , Zhang, X. , McCarthy, J. J. , McDearmon, E. L. , Hornberger, T. A. , Russell, B. , Campbell, K. S. , Arbogast, S. , Reid, M. B. , Walker, J. R. , Hogenesch, J. B. , Takahashi, J. S. , & Esser, K. A. (2010). CLOCK and BMAL1 regulate MyoD and are necessary for maintenance of skeletal muscle phenotype and function. Proceedings of the National Academy of Sciences of the United States of America, 107, 19090–19095. 10.1073/pnas.1014523107 20956306 PMC2973897

[phy270902-bib-0005] Brooks, S. V. , & Faulkner, J. A. (1988). Contractile properties of skeletal muscles from young, adult and aged mice. The Journal of Physiology, 404, 71–82. 10.1113/jphysiol.1988.sp017279 3253447 PMC1190815

[phy270902-bib-0006] Brooks, S. V. , Faulkner, J. A. , & McCubbrey, D. A. (1990). Power outputs of slow and fast skeletal muscles of mice. Journal of Applied Physiology (Bethesda, MD: 1985), 68, 1282–1285. 10.1152/jappl.1990.68.3.1282 2341351

[phy270902-bib-0007] Burkholder, T. J. , Fingado, B. , Baron, S. , & Lieber, R. L. (1994). Relationship between muscle fiber types and sizes and muscle architectural properties in the mouse hindlimb. Journal of Morphology, 221, 177–190. 10.1002/jmor.1052210207 7932768

[phy270902-bib-0008] Chapman, M. A. , Zhang, J. , Banerjee, I. , Guo, L. T. , Zhang, Z. , Shelton, G. D. , Ouyang, K. , Lieber, R. L. , & Chen, J. (2014). Disruption of both nesprin 1 and desmin results in nuclear anchorage defects and fibrosis in skeletal muscle. Human Molecular Genetics, 23, 5879–5892. 10.1093/hmg/ddu310 24943590 PMC4204769

[phy270902-bib-0009] Claflin, D. R. , & Faulkner, J. A. (1985). Shortening velocity extrapolated to zero load and unloaded shortening velocity of whole rat skeletal muscle. The Journal of Physiology, 359, 357–363. 10.1113/jphysiol.1985.sp015589 3999042 PMC1193379

[phy270902-bib-0010] Curtin, N. A. , & Edman, K. A. (1994). Force‐velocity relation for frog muscle fibres: Effects of moderate fatigue and of intracellular acidification. The Journal of Physiology, 475, 483–494. 10.1113/jphysiol.1994.sp020087 8006830 PMC1160399

[phy270902-bib-0011] Douglas, C. M. , Hesketh, S. J. , & Esser, K. A. (2021). Time of day and muscle strength: A circadian output? Physiology, 36, 44–51. 10.1152/physiol.00030.2020 33325817 PMC8425416

[phy270902-bib-0012] Dyar, K. A. , Ciciliot, S. , Wright, L. E. , Biensø, R. S. , Tagliazucchi, G. M. , Patel, V. R. , Forcato, M. , Paz, M. I. P. , Gudiksen, A. , Solagna, F. , Albiero, M. , Moretti, I. , Eckel‐Mahan, K. L. , Baldi, P. , Sassone‐Corsi, P. , Rizzuto, R. , Bicciato, S. , Pilegaard, H. , Blaauw, B. , & Schiaffino, S. (2014). Muscle insulin sensitivity and glucose metabolism are controlled by the intrinsic muscle clock. Molecular Metabolism, 3, 29–41. 10.1016/j.molmet.2013.10.005 24567902 PMC3929910

[phy270902-bib-0013] Ezagouri, S. , Zwighaft, Z. , Sobel, J. , Baillieul, S. , Doutreleau, S. , Ladeuix, B. , Golik, M. , Verges, S. , & Asher, G. (2019). Physiological and molecular dissection of daily variance in exercise capacity. Cell Metabolism, 30, 78–91. 10.1016/j.cmet.2019.03.012 31006590

[phy270902-bib-0014] Fitzgerald, L. S. , Bremner, S. N. , Ward, S. R. , Cho, Y. , & Schenk, S. (2024). Intrinsic skeletal muscle function and contraction‐stimulated glucose uptake do not vary by time‐of‐day in mice. Function (Oxford), 5, zqae035. 10.1093/function/zqae035 PMC11873798 39134511

[phy270902-bib-0015] Fry, C. S. , Lee, J. D. , Mula, J. , Kirby, T. J. , Jackson, J. R. , Liu, F. , Yang, L. , Mendias, C. L. , Dupont‐Versteegden, E. E. , McCarthy, J. J. , & Peterson, C. A. (2015). Inducible depletion of satellite cells in adult, sedentary mice impairs muscle regenerative capacity but does not contribute to sarcopenia. Nature Medicine, 21, 76–80. 10.1038/nm.3710 PMC4289085 25501907

[phy270902-bib-0016] Glenmark, B. , Nilsson, M. , Gao, H. , Gustafsson, J.‐A. , Dahlman‐Wright, K. , & Westerblad, H. (2004). Difference in skeletal muscle function in males vs. females: Role of estrogen receptor‐beta. American Journal of Physiology. Endocrinology and Metabolism, 287, E1125–E1131. 10.1152/ajpendo.00098.2004 15280152

[phy270902-bib-0017] Haroon, M. , Klein‐Nulend, J. , Bakker, A. D. , Jin, J. , Seddiqi, H. , Offringa, C. , de Wit, G. M. J. , Le Grand, F. , Giordani, L. , Liu, K. J. , Knight, R. D. , & Jaspers, R. T. (2021). Myofiber stretch induces tensile and shear deformation of muscle stem cells in their native niche. Biophysical Journal, 120, 2665–2678. 10.1016/j.bpj.2021.05.021 34087215 PMC8390894

[phy270902-bib-0018] Hill, A. V. (1938). The heat of shortening and the dynamic constants of muscle. Proceedings of the Biological Sciences, 126, 136–195. 10.1098/rspb.1938.0050

[phy270902-bib-0019] Hill, A. V. (1953). The mechanics of active muscle. Proceedings of the Royal Society of London‐Series B: Biological Sciences, 141, 104–117. 10.1098/rspb.1953.0027 13047276

[phy270902-bib-0020] Hill, C. , James, R. S. , Cox, V. M. , Seebacher, F. , & Tallis, J. (2020). Age‐related changes in isolated mouse skeletal muscle function are dependent on sex, muscle, and contractility mode. American Journal of Physiology. Regulatory, Integrative and Comparative Physiology, 319, R296–R314. 10.1152/ajpregu.00073.2020 32697655

[phy270902-bib-0021] Kahn, R. E. , Dinnunhan, F. , Meza, G. , Lieber, R. L. , Lacham‐Kaplan, O. , Hawley, J. A. , & Dayanidhi, S. (2025). Time‐of‐day effects on muscle mitochondria following short‐term ablation of satellite cells. Frontiers in Physiology, 16, 1613184. 10.3389/fphys.2025.1613184 40671712 PMC12263925

[phy270902-bib-0022] Kahn, R. E. , Krater, T. , Larson, J. E. , Encarnacion, M. , Karakostas, T. , Patel, N. M. , Swaroop, V. T. , & Dayanidhi, S. (2023). Resident muscle stem cell myogenic characteristics in postnatal muscle growth impairments in children with cerebral palsy. American Journal of Physiology‐Cell Physiology, 324, C614–C631. 10.1152/ajpcell.00499.2022 36622072 PMC9942895

[phy270902-bib-0023] Kahn, R. E. , Lieber, R. L. , Meza, G. , Dinnunhan, F. , Lacham‐Kaplan, O. , Dayanidhi, S. , & Hawley, J. A. (2024). Time‐of‐day effects on ex vivo muscle contractility following short‐term satellite cell ablation. American Journal of Physiology‐Cell Physiology, 327, C213–C219. 10.1152/ajpcell.00157.2024 38586876 PMC11371314

[phy270902-bib-0024] Kahn, R. E. , Zhu, P. , Roy, I. , Peek, C. , Hawley, J. A. , & Dayanidhi, S. (2025). Ablation of satellite cell‐specific clock gene, Bmal1, alters force production, muscle damage, and repair following contractile‐induced injury. The FASEB Journal, 39, e70325. 10.1096/fj.202402145RR 39812604 PMC11734708

[phy270902-bib-0025] Kann, A. P. , Hung, M. , Wang, W. , Nguyen, J. , Gilbert, P. M. , Wu, Z. , & Krauss, R. S. (2022). An injury‐responsive Rac‐to‐rho GTPase switch drives activation of muscle stem cells through rapid cytoskeletal remodeling. Cell Stem Cell, 29, 933–947. 10.1016/j.stem.2022.04.016 35597234 PMC9177759

[phy270902-bib-0026] Kemler, D. , Wolff, C. A. , & Esser, K. A. (2020). Time‐of‐day dependent effects of contractile activity on the phase of the skeletal muscle clock. The Journal of Physiology, 598, 3631–3644. 10.1113/JP279779 32537739 PMC7479806

[phy270902-bib-0027] Kinney, M. C. , Dayanidhi, S. , Dykstra, P. B. , McCarthy, J. J. , Peterson, C. A. , & Lieber, R. L. (2017). Reduced skeletal muscle satellite cell number alters muscle morphology after chronic stretch but allows limited serial sarcomere addition. Muscle & Nerve, 55, 384–392. 10.1002/mus.25227 27343167 PMC5183525

[phy270902-bib-0028] Kumar, A. , Vaca‐Dempere, M. , Mortimer, T. , Deryagin, O. , Smith, J. G. , Petrus, P. , Koronowski, K. B. , Greco, C. M. , Segalés, J. , Andrés, E. , Lukesova, V. , Zinna, V. M. , Welz, P.‐S. , Serrano, A. L. , Perdiguero, E. , Sassone‐Corsi, P. , Benitah, S. A. , & Muñoz‐Cánoves, P. (2024). Brain‐muscle communication prevents muscle aging by maintaining daily physiology. Science, 384, 563–572. 10.1126/science.adj8533 38696572

[phy270902-bib-0029] Lännergren, J. , & Westerblad, H. (1991). Force decline due to fatigue and intracellular acidification in isolated fibres from mouse skeletal muscle. The Journal of Physiology, 434, 307–322. 10.1113/jphysiol.1991.sp018471 1902515 PMC1181419

[phy270902-bib-0030] Lutz, G. J. , & Lieber, R. L. (2000). Myosin isoforms in anuran skeletal muscle: Their influence on contractile properties and in vivo muscle function. Microscopy Research and Technique, 50, 443–457. 10.1002/1097-0029(20000915)50:6<443::AID-JEMT3>3.0.CO;2-5 10998635

[phy270902-bib-0031] Lutz, G. J. , Sirsi, S. R. , Shapard‐Palmer, S. A. , Bremner, S. N. , & Lieber, R. L. (2002). Influence of myosin isoforms on contractile properties of intact muscle fibers from Rana pipiens. American Journal of Physiology. Cell Physiology, 282, C835–C844. 10.1152/ajpcell.00482.2001 11880272

[phy270902-bib-0032] Maier, G. , Delezie, J. , Westermark, P. O. , Santos, G. , Ritz, D. , & Handschin, C. (2022). Transcriptomic, proteomic and phosphoproteomic underpinnings of daily exercise performance and zeitgeber activity of training in mouse muscle. The Journal of Physiology, 600, 769–796. 10.1113/JP281535 34142717 PMC9290843

[phy270902-bib-0033] Mayeuf‐Louchart, A. , Hardy, D. , Thorel, Q. , Roux, P. , Gueniot, L. , Briand, D. , Mazeraud, A. , Bouglé, A. , Shorte, S. L. , Staels, B. , Chrétien, F. , Duez, H. , & Danckaert, A. (2018). MuscleJ: A high‐content analysis method to study skeletal muscle with a new Fiji tool. Skeletal Muscle, 8, 25. 10.1186/s13395-018-0171-0 30081940 PMC6091189

[phy270902-bib-0034] McCarthy, J. J. , Andrews, J. L. , McDearmon, E. L. , Campbell, K. S. , Barber, B. K. , Miller, B. H. , Walker, J. R. , Hogenesch, J. B. , Takahashi, J. S. , & Esser, K. A. (2007). Identification of the circadian transcriptome in adult mouse skeletal muscle. Physiological Genomics, 31, 86–95. 10.1152/physiolgenomics.00066.2007 17550994 PMC6080860

[phy270902-bib-0035] McCarthy, J. J. , Mula, J. , Miyazaki, M. , Erfani, R. , Garrison, K. , Farooqui, A. B. , Srikuea, R. , Lawson, B. A. , Grimes, B. , Keller, C. , Van Zant, G. , Campbell, K. S. , Esser, K. A. , Dupont‐Versteegden, E. E. , & Peterson, C. A. (2011). Effective fiber hypertrophy in satellite cell‐depleted skeletal muscle. Development, 138, 3657–3666. 10.1242/dev.068858 21828094 PMC3152923

[phy270902-bib-0036] Méndez, J. , & Keys, A. (2026). Density and composition of mammalian muscle. https://www.semanticscholar.org/paper/Density‐and‐composition‐of‐mammalian‐muscle‐M%C3%A9ndez‐Keys/9ae28a7ad4ce13efb0cf7e6cfb32b6d222b9208b

[phy270902-bib-0037] Miller, B. H. , McDearmon, E. L. , Panda, S. , Hayes, K. R. , Zhang, J. , Andrews, J. L. , Antoch, M. P. , Walker, J. R. , Esser, K. A. , Hogenesch, J. B. , & Takahashi, J. S. (2007). Circadian and CLOCK‐controlled regulation of the mouse transcriptome and cell proliferation. Proceedings of the National Academy of Sciences of the United States of America, 104, 3342–3347. 10.1073/pnas.0611724104 17360649 PMC1802006

[phy270902-bib-0038] Murach, K. A. , White, S. H. , Wen, Y. , Ho, A. , Dupont‐Versteegden, E. E. , McCarthy, J. J. , & Peterson, C. A. (2017). Differential requirement for satellite cells during overload‐induced muscle hypertrophy in growing versus mature mice. Skeletal Muscle, 7, 14. 10.1186/s13395-017-0132-z 28693603 PMC5504676

[phy270902-bib-0039] Palmisano, M. G. , Bremner, S. N. , Hornberger, T. A. , Meyer, G. A. , Domenighetti, A. A. , Shah, S. B. , Kiss, B. , Kellermayer, M. , Ryan, A. F. , & Lieber, R. L. (2015). Skeletal muscle intermediate filaments form a stress‐transmitting and stress‐signaling network. Journal of Cell Science, 128, 219–224. 10.1242/jcs.142463 25413344 PMC4294770

[phy270902-bib-0040] Reilly, T. , & Brooks, G. A. (1982). Investigation of circadian rhythms in metabolic responses to exercise. Ergonomics, 25, 1093–1107. 10.1080/00140138208925067 7173159

[phy270902-bib-0041] Rome, L. C. (2006). Design and function of superfast muscles: New insights into the physiology of skeletal muscle. Annual Review of Physiology, 68, 193–221. 10.1146/annurev.physiol.68.040104.105418 16460271

[phy270902-bib-0042] Sam, M. , Shah, S. , Fridén, J. , Milner, D. J. , Capetanaki, Y. , & Lieber, R. L. (2000). Desmin knockout muscles generate lower stress and are less vulnerable to injury compared with wild‐type muscles. American Journal of Physiology. Cell Physiology, 279, C1116–C1122. 10.1152/ajpcell.2000.279.4.C1116 11003592

[phy270902-bib-0043] Schroder, E. A. , Harfmann, B. D. , Zhang, X. , Srikuea, R. , England, J. H. , Hodge, B. A. , Wen, Y. , Riley, L. A. , Yu, Q. , Christie, A. , Smith, J. D. , Seward, T. , Wolf Horrell, E. M. , Mula, J. , Peterson, C. A. , Butterfield, T. A. , & Esser, K. A. (2015). Intrinsic muscle clock is necessary for musculoskeletal health. The Journal of Physiology, 593, 5387–5404. 10.1113/JP271436 26486627 PMC4704520

[phy270902-bib-0044] Small, L. , Altıntaş, A. , Laker, R. C. , Ehrlich, A. , Pattamaprapanont, P. , Villarroel, J. , Pillon, N. J. , Zierath, J. R. , & Barrès, R. (2020). Contraction influences Per2 gene expression in skeletal muscle through a calcium‐dependent pathway. The Journal of Physiology, 598, 5739–5752. 10.1113/JP280428 32939754 PMC7756801

[phy270902-bib-0045] Smith, J. A. B. , Murach, K. A. , Dyar, K. A. , & Zierath, J. R. (2023). Exercise metabolism and adaptation in skeletal muscle. Nature Reviews. Molecular Cell Biology, 24, 607–632. 10.1038/s41580-023-00606-x 37225892 PMC10527431

[phy270902-bib-0046] Solanas, G. , Peixoto, F. O. , Perdiguero, E. , Jardí, M. , Ruiz‐Bonilla, V. , Datta, D. , Symeonidi, A. , Castellanos, A. , Welz, P.‐S. , Caballero, J. M. , Sassone‐Corsi, P. , Muñoz‐Cánoves, P. , & Benitah, S. A. (2017). Aged stem cells reprogram their daily rhythmic functions to adapt to stress. Cell, 170, 678–692. 10.1016/j.cell.2017.07.035 28802040

[phy270902-bib-0047] Tavi, P. , Korhonen, T. , Hänninen, S. L. , Bruton, J. D. , Lööf, S. , Simon, A. , & Westerblad, H. (2010). Myogenic skeletal muscle satellite cells communicate by tunnelling nanotubes. Journal of Cellular Physiology, 223, 376–383. 10.1002/jcp.22044 20112291

[phy270902-bib-0048] Wolff, G. , & Esser, K. A. (2012). Scheduled exercise phase shifts the circadian clock in skeletal muscle. Medicine and Science in Sports and Exercise, 44, 1663–1670. 10.1249/MSS.0b013e318255cf4c 22460470 PMC3414645

[phy270902-bib-0049] Zhu, P. , Pfrender, E. M. , Steffeck, A. W. T. , Reczek, C. R. , Zhou, Y. , Thakkar, A. V. , Gupta, N. R. , Kupai, A. , Willbanks, A. , Lieber, R. L. , Roy, I. , Chandel, N. S. , & Peek, C. B. (2025). Immunomodulatory role of the stem cell circadian clock in muscle repair. Science Advances, 11, eadq8538. 10.1126/sciadv.adq8538 40043110 PMC11881903

