# Supplementary figures and images for: Time‐of‐day, satellite cells, and velocity collectively influence ex vivo isovelocity force production in mouse extensor digitorum longus muscle
Source: Physiol Rep. 2026 May 3;14(9):e70902. doi: 10.14814/phy2.70902 (PMC13136071; doi:10.14814/phy2.70902)

**S1A**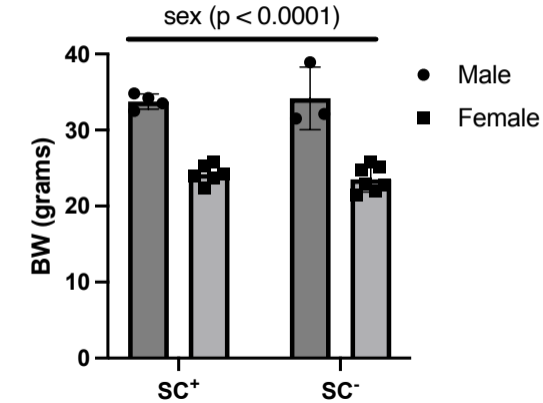**S1B**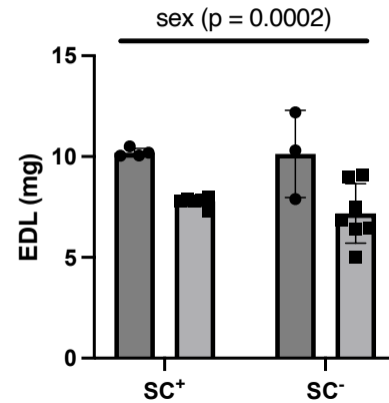**S1C**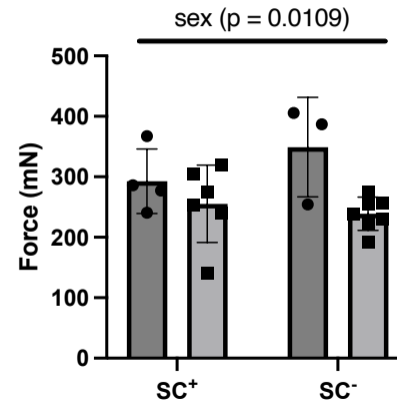**S1D**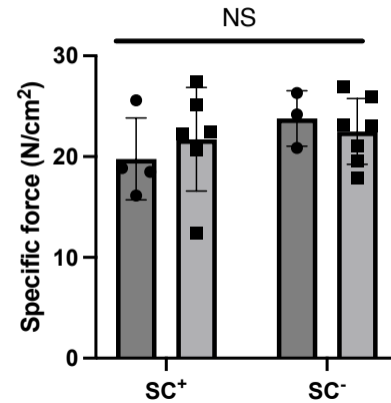

Supplement: Supplementary file 1 — Figure S1. Sex comparisons of BW, EDL weight, force, and specific force. (A–D) Sex comparisons in BW (p < 0.0001), EDL weight (p = 0.0002), force (p = 0.0109), and specific force (NS) in both SC+ and SC− groups. All data shown as mean ± SD. All groups compared via two‐way ANOVA (n = 3–5 mouse EDLs per group). [file PHY2-14-e70902-s001.pdf]
